# Targeting of SET/I2PP2A oncoprotein inhibits Gli1 transcription revealing a new modulator of Hedgehog signaling

**DOI:** 10.1038/s41598-021-93440-0

**Published:** 2021-07-06

**Authors:** Iliana Serifi, Simoni Besta, Zoe Karetsou, Panagiota Giardoglou, Dimitris Beis, Pawel Niewiadomski, Thomais Papamarcaki

**Affiliations:** 1 Laboratory of Biological Chemistry, Medical Department, School of Health Sciences, University of Ioannina, 451 10 Ioannina, Greece; 2 Department of Biomedical Research, Foundation for Research and Technology-Hellas, Institute of Molecular Biology and Biotechnology, 451 10 Ioannina, Greece; 3 Developmental Biology, Center for Clinical, Experimental Surgery and Translational Research, Biomedical Research Foundation Academy of Athens, 115 27 Athens, Greece; 4 Centre of New Technologies, University of Warsaw, Warsaw, Poland

**Keywords:** Cell biology, Developmental biology, Molecular biology

## Abstract

The Hedgehog (Hh)/Gli signaling pathway controls cell proliferation and differentiation, is critical for the development of nearly every tissue and organ in vertebrates and is also involved in tumorigenesis. In this study, we characterize the oncoprotein SET/I2PP2A as a novel regulator of Hh signaling. Our previous work has shown that the zebrafish homologs of SET are expressed during early development and localized in the ciliated organs. In the present work, we show that CRISPR/Cas9-mediated knockdown of *setb* gene in zebrafish embryos resulted in cyclopia, a characteristic patterning defect previously reported in Hh mutants. Consistent with these findings, targeting *setb* gene using CRISPR/Cas9 or a *setb* morpholino, reduced Gli1-dependent mCherry expression in the Hedgehog reporter zebrafish line Tg(12xGliBS:mCherry-NLS). Likewise, SET loss of function by means of pharmacological inhibition and gene knockdown prevented the increase of Gli1 expression in mammalian cells in vitro. Conversely, overexpression of SET resulted in an increase of the expression of a Gli-dependent luciferase reporter, an effect likely attributable to the relief of the Sufu-mediated inhibition of Gli1. Collectively, our data support the involvement of SET in Gli1-mediated transcription and suggest the oncoprotein SET/I2PP2A as a new modulator of Hedgehog signaling.

## Introduction

The Hedgehog (Hh) signaling pathway controls cell proliferation and differentiation and is critical for the development of nearly every tissue and organ in vertebrates^1^. Lack of Hh signaling during development is embryonic lethal, while hyperactivation of the Hh pathway promotes tumor formation and maintenance of a wide range of human malignancies, including medulloblastoma and basal cell carcinoma of the skin^2^. Several findings suggest that Hh signaling might also modulate the tumor microenvironment, which has important implications for drug screening and therapeutic interventions^3^. Therefore, Hh signal transduction has emerged as a key pathway for cancer therapy and its pharmacological modulation is of great importance in clinical trials^4^.

Unlike other signaling pathways involved in development, the vertebrate Hh signaling is dependent on the primary cilium, a microtubule-based membrane protrusion that functions as an antenna in the cells^5^. The main effectors of the pathway are Gli transcription factors (Gli1, Gli2, Gli3) which belong to the Krüppel family of zinc finger proteins. Gli1 and Gli2 are positive regulators of transcription, whereas Gli3 mainly functions as a transcription repressor^6^. Upon their transportation into the nucleus, Gli transcription factors activate target genes that participate in the regulation of proliferation and self-renewal^7,8^. The localization and the activity of Gli transcription factors, control both the amplitude and the outcome of the Hh expression program, especially during development, and are regulated by a complex combination of posttranslational modifications, including phosphorylation^8,9^, acetylation^10^ and ubiquitination/proteasomal truncation^11^.

The oncoprotein SET is mainly a nuclear protein that functions as a histone chaperone^12,13^, transcription cofactor^14–16^, regulator of histone acetylation^17,18^, modulator of DNA repair^19^ and as an epigenetic regulator^20^. Besides its nuclear function, SET is an inhibitor of protein phosphatase 2A (PP2A)^21^ and has also been reported as a signaling amplifier of Rac1-dependent cell migration^22^.

SET was first characterized in acute undifferentiated leukemia^23^ and is overexpressed in various cancers, including lymphoid malignancies^24^ and non-small cell lung carcinoma (NSCLC)^25^. In addition, SET is involved in the epithelial mesenchymal transition (EMT)^26^ and in the regulation of cancer cell stemness^27^.

Several studies have focused on SET targeting as a potential therapeutic approach in cancer^28,29^, by testing the effects of two SET inhibitors, COG112^28,30^ and FTY720^24,31–33^ on cancer progression. COG112 is an apolipoprotein E (apoE)-mimetic peptide which inhibits SET-Rac1 interaction resulting to decreased cell migration and invasion of cancer cells^28,30^. FTY720 (Fingolimod) is a sphingosine immunosuppressant drug that also mediates the suppression of several tumors^24,31–33^. Recent work has reported impairment of triple negative breast cancer progression upon blockage of SET function by TD19, a new compound which is a derivative of erlotinib^34^. Besides the pharmacological inhibition of SET, other studies have shown that knocking down SET in Smo1 primary medulloblastoma cells induces cell death, suggesting a potential function of the oncoprotein in Hh signaling^35^. In this study, we present several pieces of evidence which support the involvement of SET in Gli1-mediated transcription that plays a crucial role in the Hh signaling cascade.

## Results

### SET is involved in Hh signaling in zebrafish

**Figure 1:**
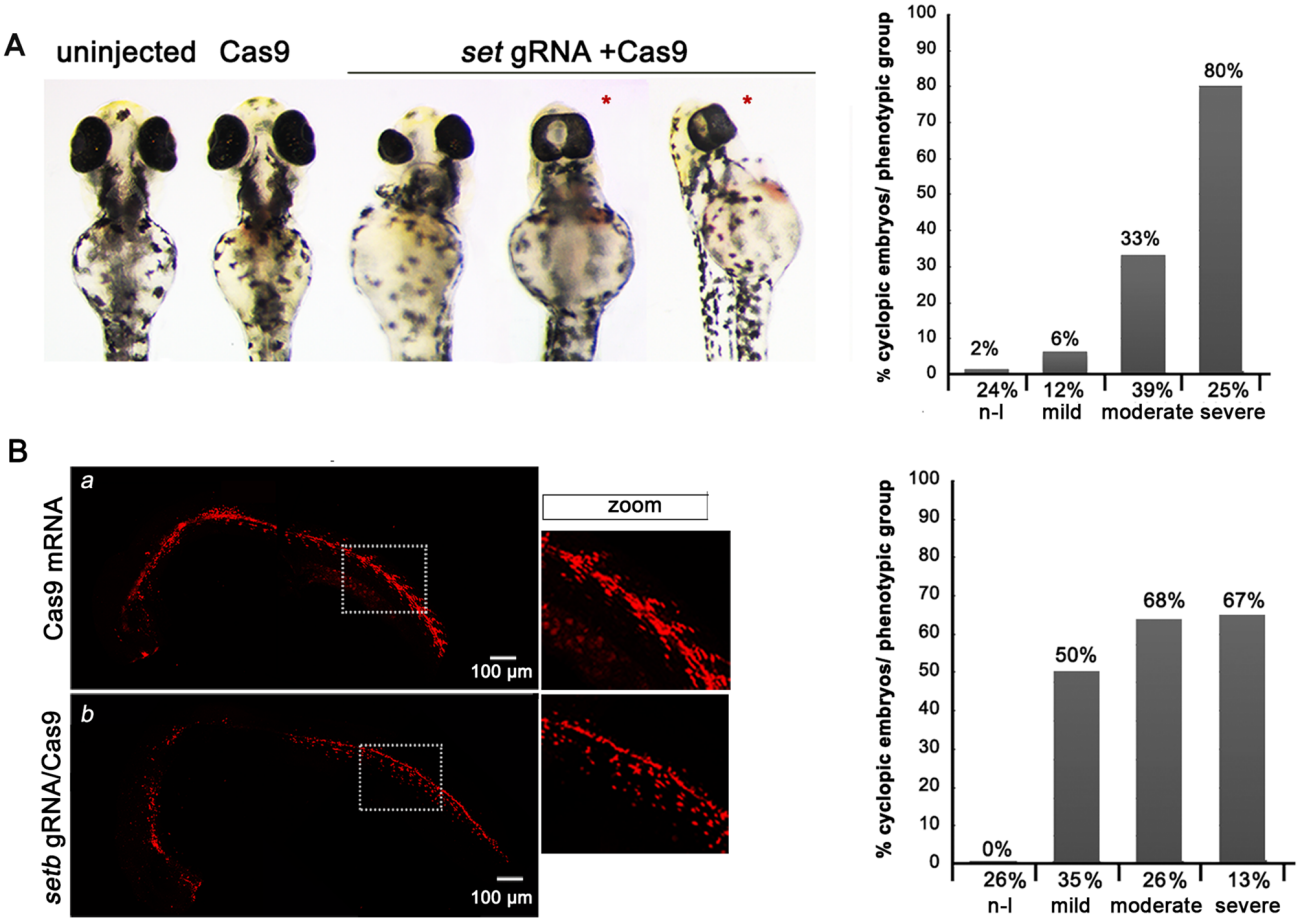
Impact of targeting the *setb* gene on Hedgehog signaling in zebrafish embryos. (**A**) Zebrafish *setb* crispants exhibit cyclopia. One- to two-cell stage zebrafish embryos were injected with 4.6 nl of 150 ng/μl of Cas9 mRNA or with 50 ng/μl of *setb* gRNA and 150 ng/μl of Cas9 mRNA and were examined morphologically at 48 hpf. (Right graph). Phenotypic scoring of the injected embryos (n = 262, n denotes the number of injected embryos). Numbers in X-axis refer to percentage of embryos in each phenotypic group and numbers on the bars refer to the percentage of cyclopic embryos in each phenotypic group. (**B**) Hh signaling is reduced in Tg12x_Gli transgenic zebrafish embryos injected with *setb* gRNA/Cas9. One- to two-cell stage Tg12x_Gli transgenic zebrafish embryos were injected with *setb* gRNA/Cas9 and examined under a confocal fluorescence microscope at 24 hpf. The phenotypic scoring of the injected embryos is shown in the graph (n = 96). The squares show enlargement of the trunk areas that display decreased mCherry fluorescence intensity. Note the disrupted morphology of the somites in the marked regions.

Previous work of our lab has shown that the zebrafish homologs of SET oncoprotein are expressed during early zebrafish development and localized in the ciliated organs of zebrafish, suggesting a potential function of SET in signaling cascades coordinated by cilia^36^. This study also demonstrated that targeting *setb* gene expression by injections of *setb* gRNA with Cas9 mRNA or *seta/b* morpholinos into one-cell stage zebrafish embryos resulted in several morphological abnormalities, including defects in spinal cord and eye development^36^. A systematic scoring of the eye defects in *setb* crispants showed that approximately 80% of the larvae with severe phenotype and 33% of the larvae with moderate phenotype exhibited cyclopia (Fig. 1A), a characteristic embryonic patterning defect reported in the Sonic Hedgehog mutant mouse and zebrafish^37,38^.

To investigate in detail the potential role of *setb* gene in Hh signaling we next used the Sonic Hedgehog reporter zebrafish line *Tg(12xGliBS:mCherry-NLS)*, which expresses the mCherry reporter under the transcriptional regulation of Gli1^39^. To this end, *setb* gRNA was coinjected with Cas9 mRNA in Tg12x_Gli embryos and the transgene expression was recorded at 24 hpf. As shown in Fig. 1B, *setb* crispants displayed a decrease of the mCherry reporter expression, mainly in the trunk region. Importantly, the somite development, a process that depends on the Hh signaling pathway, was disturbed in the injected embryos, as shown by the defective segmentation of the somites (Fig. 1B). Similar results were obtained upon targeting both *seta* and *setb* genes in the transgenic embryos with a translation blocking morpholino (MOab)^36^ (Fig. 1S).

**Figure 2:**
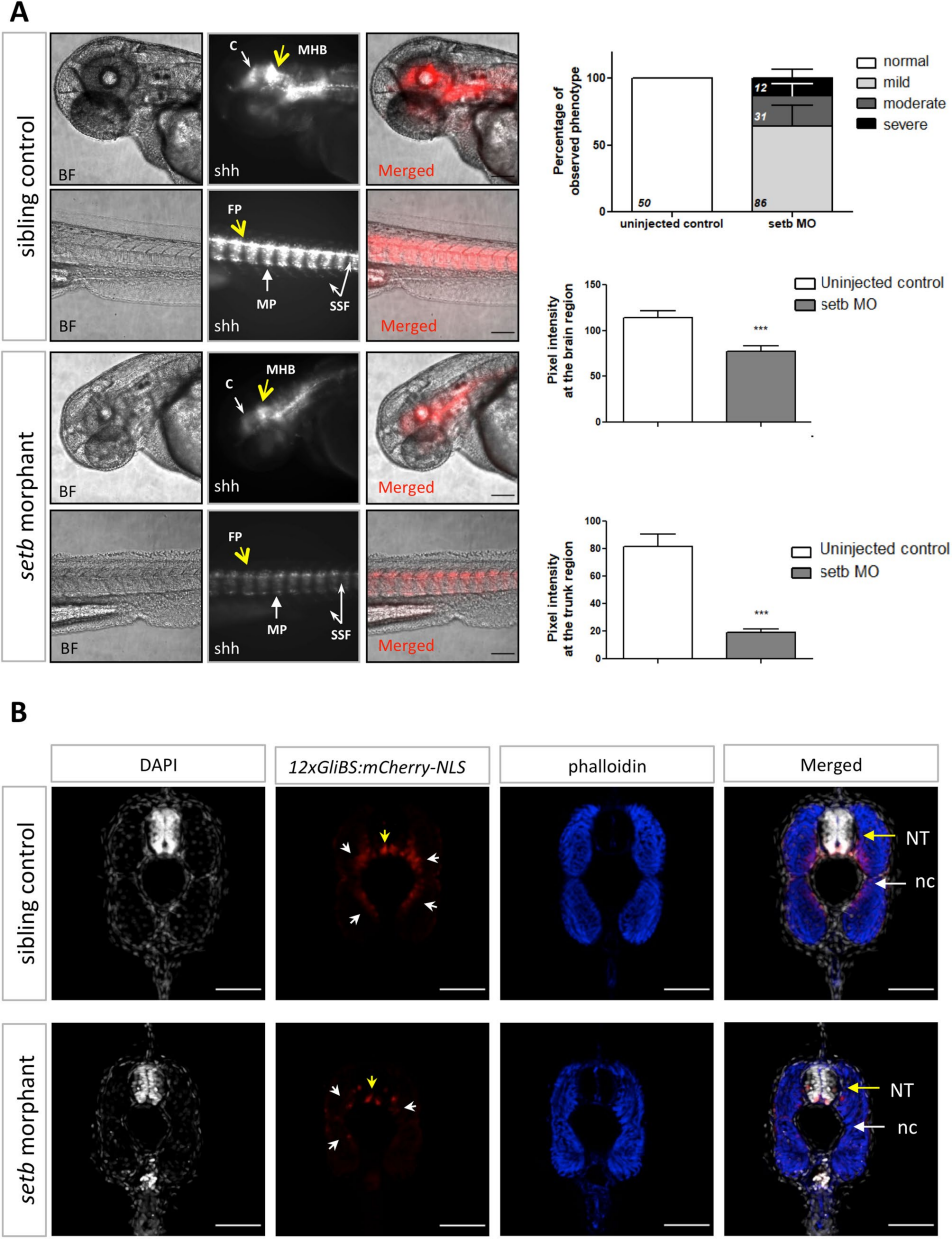
Activation of Hh signaling is reduced in *setb* morphants*.* (**A**) Fluorescent and brightfield images of 48 hpf *setb*-morpholino injected and control sibling Tg12x_Gli embryos. Embryos are placed anterior to left, dorsal up. Scale bars 100 μm. Quantification of pixel intensity at 48 hpf of the morphologically mildly affected embryos (***p < 0.001). Data are expressed as mean ± SEM (n = 129, after exclusion of the injected embryos developing apoptotic tissue at 24 hpf). Percentage of phenotypic scoring of 48 hpf morphants is presented with mean ± SEM and the numbers of total embryos per group is indicated. At the brain region: cerebellum (c, white arrow), mid-hindbrain boundary (MHB, yellow arrow). At the trunk region: floor plate (FP, yellow arrow), slow muscle pioneer cells (MP, white arrow, high mCherry signal), superficial slow fibers (SSF, white arrows, mid mCherry signal). (**B**) Confocal images of cross-sections throughout the trunk region of 48 hpf *setb* morphants and age-matched siblings. Note the reduction of mCherry^+^ cells at the ventral side of neural tube (yellow arrows) and at the slow fibers surrounding notochord (white arrows) of *setb* morphants; indication of lower activation levels of the Hh pathway. Sections were stained with phalloidin-633 (blue) and DAPI (grey). NT: neural tube, nc: notochord. Scale bars 50 μm.

In parallel, we performed knockdown experiments to block the *setb* function by injecting one-cell stage Tg12x_Gli zebrafish embryos with antisense morpholino oligonucleotides targeting the *setb* gene. *setb* morphants exhibited reduced mCherry reporter activity compared to age-matched siblings, indicating lower levels of Hh signaling activation. In accordance with the *setb* crispants, the morphants appear to show decreased mCherry fluorescence in both the brain and the trunk area (ventral neural tube and conformation of somites) (Fig. 2A). In order to analyze at cellular resolution the reduction of Hh signaling at the neural tube of *setb* morphants, 48 hpf larvae were sectioned transversally. Hh-activated cells (mCherry^+^) both at the floor plate of neural tube and the slow fibers adjacent to the notochord exhibited significant signal reduction compared to the control siblings (Fig. 2B). These data suggest the involvement of *set* genes in the regulation of Hh signaling during zebrafish development.

### Targeting SET in NIH 3T3 cells inhibits Hh signaling

**Figure 3:**
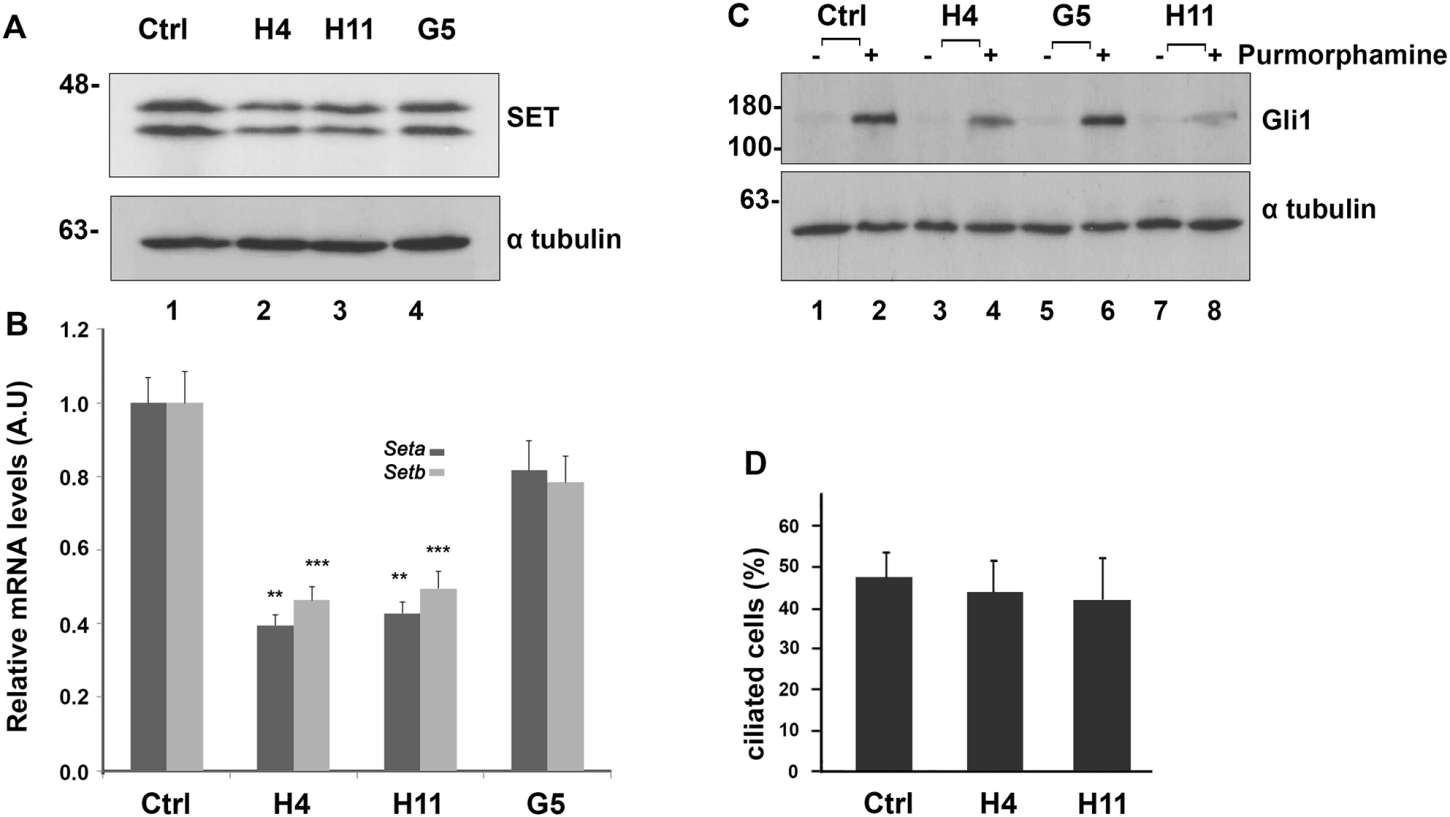
CRISPR/Cas9-mediated SETKD is linked with a decrease in Gli1 expression. (**A**) SET protein levels in NIH 3T3 control cells and SETKD clones. Western blot analysis of control NIH 3T3, H4, H11 and G5 cell extracts using the anti-I2PP2A and anti-α-tubulin antibodies (loading control). Original uncropped Western Blot images are shown in Supplementary Fig. 2S. (**B**) Comparison of the relative expression differences for *Seta* and *Setb*, the two splicing forms of mouse *Set* gene, in control NIH 3T3, H4, H11 and G5 cells. The fold changes in qPCR were normalized to *Gapdh*. Data are expressed as mean ± SEM (n = 3 ) **p < 0.01***p < 0.001. (**C**) Gli1 expression levels in control NIH 3T3 and SETKD cells. Western blot analysis of extracts obtained from control NIH 3T3 cells and H4, H11 and G5 cells after incubation at 0.5% serum with DMSO (lanes 1, 3, 5, 7) or 2 μM purmorphamine (lanes 2, 4, 6, 8) using the anti-Gli1 and anti-α-tubulin antibodies. Original uncropped Western Blot images are shown in Supplementary Fig. 2S. (**D**) Ciliation analysis of control NIH 3T3 cells and H4, H11 clones after 24 h of serum starvation. 100–150 cells were analyzed per experiment (n = 3).

To further study the role of the oncoprotein SET in the Hh pathway, we used NIH 3T3 cells, a well validated Hh-responsive cell line. To this end, we established SET knock down (SETKD) cell lines by utilizing the CRISPR/Cas9 technology. A plasmid expressing a small guide RNA targeting exon 4 of the mouse *Set* gene was cotransfected with Cas9 mRNA. *Set* mRNA and protein levels were analyzed in different clones to verify the ablation of the protein. As shown in Fig. 3A, H4 and H11 clones exhibited reduced SET levels (lanes 2, 3), while the G5 clone displayed SET expression comparable to control cells (lane 4). These results were verified by qPCR analysis of SETKD clones which showed that *Set* gene expression was knocked down in the H4 and H11 clones. The generation of multiple *Set* knockdown clones and no clone with complete SET loss in our CRISPR experiment may indicate that mouse *Set* genes are essential and therefore their complete knockout was not feasible. Nevertheless, SETKD cell lines represent a cell-based experimental system complementary to zebrafish morphants and crispants that also express reduced protein levels of the zebrafish Seta/b proteins^36^.

We next asked whether the stimulated expression of Gli1 transcription factor, the constitutive activator of Hh signaling, was affected in the H4 and H11 clones. Upon treatment of control NIH 3T3 cells with purmorphamine, a specific agonist of Smo, the expression of Gli1 was increased, as expected (Fig. 3C, lanes 1, 2). Interestingly, the decrease of SET protein levels in H4 and H11 SETKD cells attenuated the stimulated Gli1 expression (Fig. 3C, lanes 4, 8), in agreement with the in vivo experiments in zebrafish (Figs. 1, 2). On the contrary, the stimulated expression of Gli1 in the G5 clone was similar to Gli1 levels of control cells (Fig. 3C, lane 6). Ciliation analysis of H4 and H11 cells showed that cilia number, a crucial structural component of Hh signaling^5^, was not affected upon the decrease of SET protein levels, indicating a direct effect of SET in Hh signaling (Fig. 3D).

**Figure 4:**
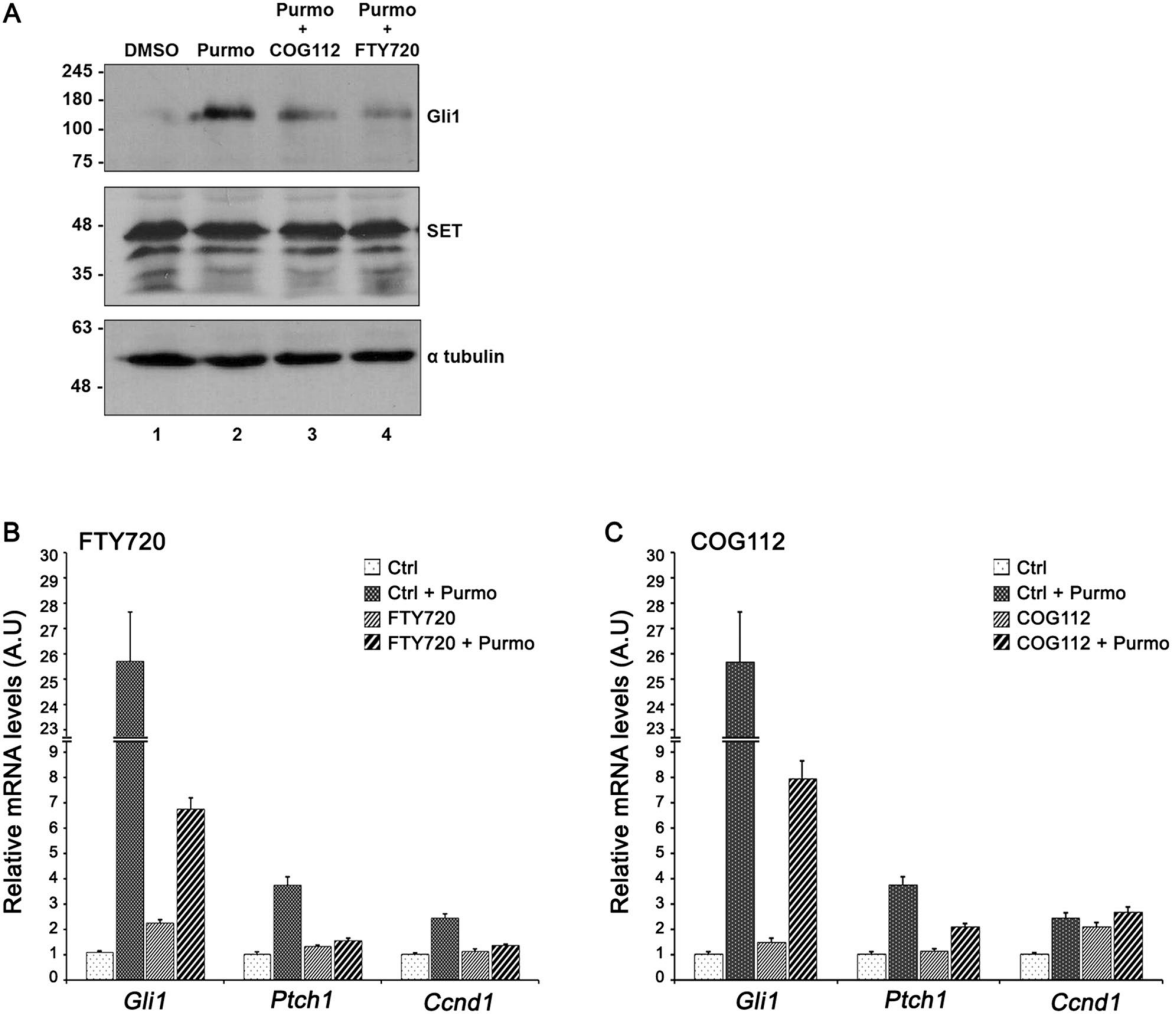
SET inhibitors decrease Gli1 expression. (**A**) The effect of FTY720 and COG112 on Gli1 protein expression. Western blot analysis of extracts obtained from NIH 3T3 cells after incubation at 0.5% serum with DMSO (lane 1) or 2 μM purmorphamine (lane 2) in the presence of 3 μM COG112 (lane 3) or 5 μM FTY720 (lane 4). Proteins were resolved in 10% SDS-PAGE and the blots were probed with the anti-Gli1, anti-SET and anti-α-tubulin antibodies (loading control). Original uncropped Western Blot images of panel A are shown in Supplementary Fig. 2S. (**B**, **C**). The effect of FTY720 and COG112 on the mRNA levels of selected genes related to Hedgehog signaling. Comparison of relative expression differences for *Gli1*, *Ptch1*, and *Ccnd1* in NIH 3T3 cells cultured at 0.5% serum after incubation with 2 µM purmorphamine in the presence of 5 μΜ FTY720 or 3 μΜ COG112, as determined by qPCR analysis. The mRNA levels of the genes were normalized to *Gapdh*. The experiments were repeated three times. Data are expressed as mean ± SEM ***p < 0.001.

In a growing number of studies, pharmacological inhibition of SET has been achieved by means of two well-studied inhibitors of SET, FTY720^24,31–33^ and COG112^28,40^. FTY720 is a sphingosine immunosuppressant drug which has been shown to have anti-tumor properties in different human malignancies^31,32^, including Hh-dependent medulloblastoma^33^. The interaction of FTY720 with SET is direct and has been studied in detail by distinct and complementary approaches, including molecular modelling^31^, biochemical binding assays^31^ and recently by NMR experiments^41^. To analyze the role of FTY720 in Hh signaling, we checked the effect of this compound in the stimulated levels of Gli1 by purmorphamine. Our results showed that treatment of the cells with 5 μΜ FTY720 prevented the increase of the stimulated levels of Gli1 (Fig. 4A, lane 4). Interestingly, treatment of the cells with COG112, an apolipoprotein E peptide ^28,40^ resulted to similar inhibition of Gli1 protein levels (Fig. 4A, lane 3). To account for differences in the temporal transcriptional response of genes involved in the Hedgehog pathway, in the presence of SET inhibitors, we performed quantitative RT-PCR analysis. Cells were cultured with 2 μΜ purmorphamine in the presence of 5 μΜ FTY720 or 3 μΜ COG112 and mRNA was isolated. As expected, upon treatment of the cells with the Hh agonist, the expression of *Gli1* and *Ptch1,* key genes that are directly stimulated by Hh signaling, was increased relative to control (Fig. 4B, C). Furthermore, the expression of the cell cycle regulator *cyclin D1* (*Ccnd1),* a Gli1 target gene, was also increased, as expected (Fig. 4B, C). Importantly, upon treatment of the cells with FTY720 or COG112, the abundance of *Gli1* and *Ptch1* mRNA was reduced in both cases, while *Ccnd1* was downregulated only in the presence of FTY720 (Fig. 4B, C). These results, taken together, suggest the involvement of SET in Hh signaling, in agreement with the data obtained from the zebrafish model system.

### SET affects Gli1-mediated transcription

**Figure 5:**
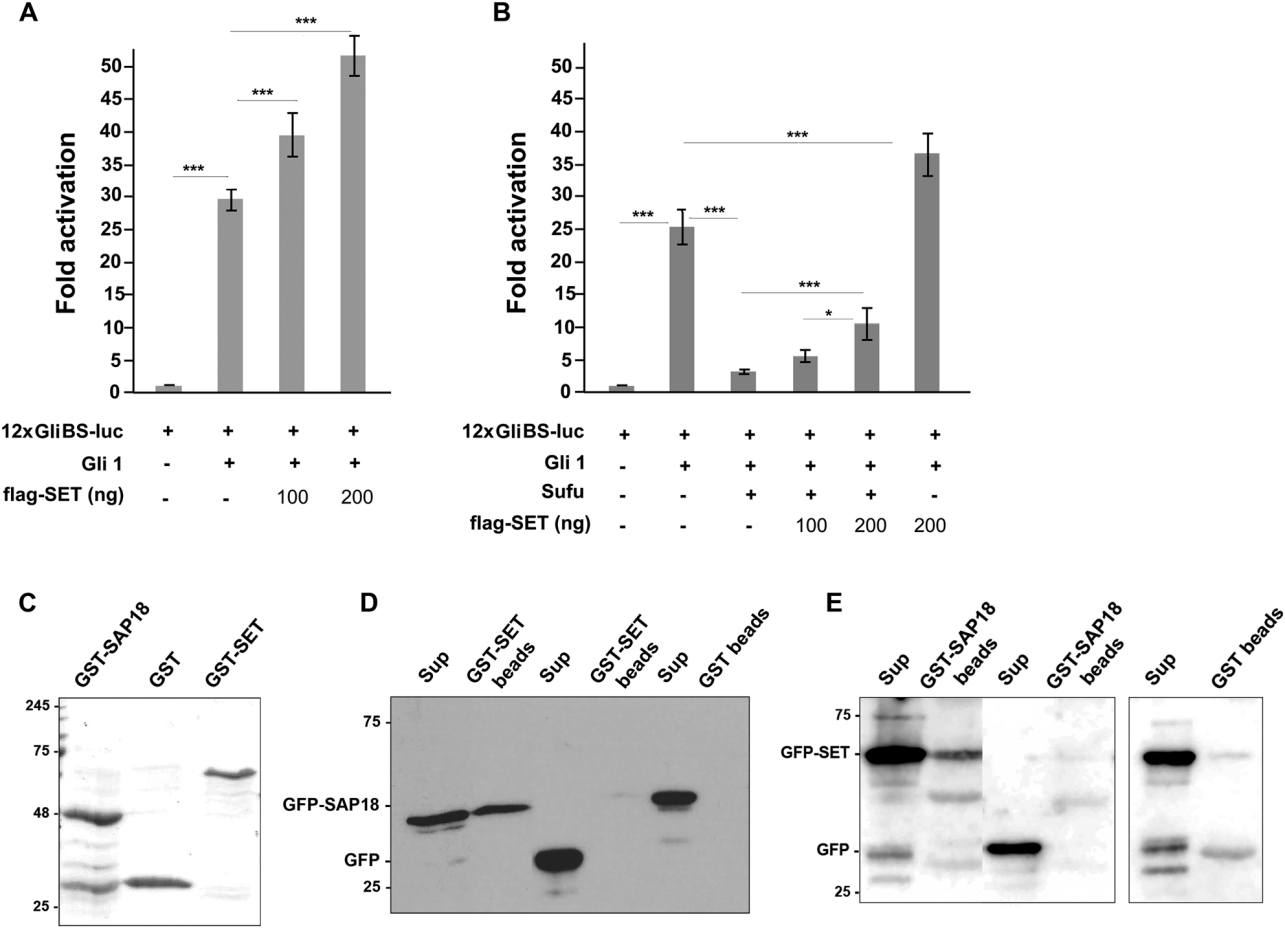
SET overexpression increases Gli1-mediated transcription. (**A**) The effect of SET on Gli1-mediated transcription. To determine Gli1-mediated transcription upon SET overexpression, 12xGliBS-luc and Gli1 expression plasmids were cotransfected with a human Flag-SET expressing construct in HEK 293 cells. (**B**) The effect of SET on Sufu inhibition of Gli1-mediated transcription. HEK 293 cells were transfected with 12xGliBS-luc reporter with the indicated amounts of plasmids encoding Gli1 (100 ng), Flag-SET (100 and 200 ng) and Sufu (10 ng). pRL-TK expresses Renilla luciferase and was included in all samples to normalize the transfection efficiency. Experiments were repeated three times and samples were analyzed in triplicates, *p < 0.05, **p < 0.01, ***p < *0.001.* (**C**) Expression of GST-SET and GST-SAP18 recombinant proteins. Bacterially expressed glutathione-S-transferase-tagged SAP18 (GST-SAP18), GST protein and baculovirus expressed GST-SET (approximately 2–3 μg each) were immobilized on glutathione-Sepharose beads and analyzed by 12% SDS-PAGE. An original uncropped image of panel C is shown in Supplementary Fig. 2S. (**D**) GST-SET pull down experiments. GST-SET or GST was immobilized on glutathione-Sepharose beads and incubated with cell extracts expressing GFP-SAP18 or GFP. (**E**) GST-SAP18 pull down experiments**.** GST-SAP18 or GST was immobilized on glutathione-Sepharose beads and incubated with cell extracts expressing GFP-SET or GFP. The beads and the supernatant of the pull down assays were analyzed by western blot using the anti-GFP antibody. Original uncropped Western Blot images of panels D and E are shown in Supplementary Fig. 2S.

Several pieces of evidence have demonstrated distinct roles of SET in gene regulation, as a transcription cofactor through interaction with CBP/p300^14,16^ and as regulator of histone acetylation^17,18^. We therefore hypothesized that SET may regulate Hh target gene expression by affecting the transcriptional activity of Gli proteins, rather than by targeting upstream pathway components, such as Patched or Smo. To test this hypothesis, we first asked whether SET could affect Gli1-mediated transcription in the absence of upstream Hh pathway activation. To this end, HEK 293 cells were transfected with a 12xGliBS luciferase construct that contains twelve copies of Gli-binding sites^42^ together with Gli1 and Flag-SET. As shown in Fig. 5A, transcription driven by the luciferase reporter was activated by Gli1, as expected. Interestingly, SET overexpression increased Gli1-mediated transcription (Fig. 5A).

It is well documented that Gli1 function is regulated by Sufu, an essential negative modulator of Hh signaling that affects both the nucleocytoplasmic localization of Gli1^7,43,44^ and the transcriptional activity of Gli1 in the nucleus, by distinct mechanisms^45–48^.

Based on this knowledge, we then asked whether SET could affect Sufu-mediated repression of Gli1 transcriptional activity. As shown in Fig. 5B, upon expression of Sufu, Gli1-mediated transcription was markedly inhibited, as previously reported^45^. Interestingly, coexpression of Flag-SET significantly relieved SuFu-mediated inhibition of Gli1 transcriptional activity (Fig. 5Β). A possible explanation for these results is that SET may modulate Sufu protein interactions, leading to increased Gli1 transcriptional activity. We tested this hypothesis by analyzing protein interactions of GFP-SET or GFP by immunoprecipitation experiments using the anti-GFP antibody, followed by mass spectrometry (MS) analysis of the immunoprecipitated proteins. This experimental approach identified SAP18 as an interactor of SET, a component of the mSin3 and histone deacetylase complex^48,49^ that cooperates with Sufu to repress Gli1-mediated transcription^45,46^. The MS analysis also revealed two known interactors of SET, glyceraldehyde-3-phosphate dehydrogenase^50^ and T-complex protein 1 subunit delta^51^. The interaction between SET and SAP18 was verified biochemically by glutathione S-transferase (GST) pull-down assays using GST-SET as bait in cell extracts expressing GFP-SAP18 or GFP protein (Fig. 5C). Consistent with the results of the mass spectrometry analysis, GFP-SAP18 was bound specifically to bead-immobilized GST-SET (Fig. 5D). The interaction of SET with SAP18 was further validated by GST-SAP18 pull down experiments in cell extracts expressing GFP-SET or GFP (Fig. 5E). These experiments provide evidence that SET interacts specifically with SAP18. Further studies should investigate the functional significance of SET-SAP18 binding with respect to Gli1 transcription at the level of Gli1-Sufu complex, taking into consideration the dynamic nature of these interactions with respect to space and time and their dependence on the cellular context.

## Discussion

The remarkable network of gene activity regulated by Hh signaling can govern cell proliferation, survival and fate, alternatives that should be fine-tuned depending on cell context and time^1^. The Hh-dependent genes involved in cell proliferation, survival and differentiation are mostly activated and correspond to a wide variety of molecular functions^52,53^. In the present study, using in vivo experiments and cell-based assays we present several pieces of evidence which demonstrate that the oncoprotein SET is involved in the Hedgehog pathway through modulation of Gli1-mediated transcription.

In our work, using the zebrafish model organism, we have shown that the *setb* crispant larvae exhibited cyclopia, a characteristic embryonic patterning defect, first reported in the Shh mutant mouse^37^ and later in zebrafish^38^. Besides the craniofacial and eye malformations, the crispants also displayed characteristic patterning defects in the spinal cord and somite development. These results are in agreement with genetic studies in zebrafish which have shown that loss of Hh signaling leads to ventral curvature of the body, deficiencies in ventral forebrain specification and spinal cord defects^6, 54^. Further evidence for the involvement of SET in the Hh pathway was obtained using the Tg12x_Gli transgenic embryos that express the mCherry reporter under the transcriptional regulation of Gli1^42^. Upon blocking the expression of *setb* gene using two different morpholinos, the *setb* splice blocking morpholino (MOb) and the translation blocking morpholino (MOab) that targets both *seta* and *setb*^36^, the transgene expression was markedly reduced. Importantly, the same effect on the transgene expression was observed in *setb* crispants. These experiments also revealed defects in somite development of both *set* morphants and *setb* crispants. The reduction of Hh signaling activation was observed in Tg12x_Gli embryos also when the overall morphology of the morphants or crispants was not severely affected, indicating that the modulation of the Hh pathway by Seta/b is one of its primary targets.

The somite morphology is an Hh-dependent process^55^ and is considered as a suitable way to diagnose impaired Hh signaling in living zebrafish embryos^56^. The first somites appear at 11 hpf on both sides of the midline and at 16 hpf the formed somites reach the tail tip and acquire the characteristic chevron shape. In contrast to wild-type embryos, Hh mutants exhibit unshaped and rounded somites^56^. Our data are also in agreement with previous studies which showed similar developmental defects upon loss of Hh activation and identified Gli1 as the main activator of Hh signaling in zebrafish^54^.

At the cellular level, and in agreement with the in vivo data from the zebrafish, the decrease of SET protein levels in SETKD cells attenuated the stimulated Gli1 expression. Similar results on Gli1 expression levels were obtained upon blocking SET function in NIH 3T3 cells with FTY720, a known inhibitor of SET^28,31,32^. Saddoughi et al.^31^ demonstrated that FTY720 directly binds SET with a Kd = 11 nM, as determined by surface plasmon resonance (SPR). Recently, the SET-FTY720 complex was characterized by NMR experiments revealing important structural information for the design of new SET inhibitors^41^. Consistent with the effect of FTY720 on Gli1 expression, upon treatment of the cells with COG112, an apolipoprotein E peptide that inhibits SET^28,40^, Gli1 expression after purmorphamine stimulation was also decreased. Future studies are required to test whether SET inhibitors also affect Hh signaling in the context of cancer.

Nowadays, ongoing research has focused on targeting the Hh pathway downstream of Smo by studying molecules that modulate Hh signaling at the level of Gli1 transcription factor^57,58^. Therefore, unraveling the underlying mechanisms by which SET affects Gli1 transcription and Hh signaling is an important task. SET is an intrinsic protein inhibitor of Protein phosphatase 2A (PP2A)^21^ and thus may regulate PP2A activity participating in the complex interplay of kinases, phosphatases and associated factors that function at different levels in the Hh pathway^8,9,11^. Genetic studies in *Drosophila* have shown that PP2A is involved in the regulation of Hh signaling^59^. In mammalian cells, an increase of PP2A activity in cancer cells led to retention of full-length Gli3 in the cytoplasm and consequently Gli3 decreased transcription activity, while Gli1 and Gli2 activities were unaffected^60^. Our preliminary experiments have shown that the protein levels of PP2A and its enzymatic activity in NIH 3T3 cells were not significantly affected upon purmorphamine activation of the Hh pathway. Therefore, future work should investigate whether SET-PP2A interaction plays a role in regulating the phosphorylation status of the Gli1 transcription factor.

At the transcription level and in the absence of upstream Hh pathway activation, SET overexpression in HEK 293 cells resulted to an increase of Gli1-mediated transcription. This result was not surprising, since several studies have shown that SET modulates chromatin status in different ways and at different levels, acting as a histone chaperone^12,13^, regulator of acetylation/deacetylation^17,18^, epigenetic cofactor^20^ and transcription coactivator through interaction with CBP/p300^14,16^. Concerning the regulation of Gli transcription factor activity, genetic studies, initially reported in *Drosophila* and later in mammalian cells, have identified CBP as an essential cofactor for Ci and Gli transcription factors^61^. Since CBP possesses histone acetyltransferase (HAT) activity, its interaction with Gli transcription factors possibly leads to epigenetic changes of Gli targets, facilitating the accessibility of other regulators to chromatin. In this context, SET may co-operate with CBP to enhance Gli1 mediated transcription of different Hh target genes. Consistent with this hypothesis, previous work has shown that the acetyltransferase PCAF interacts with Gli1 and regulates acetylation of histone H3 on Hh target gene promoters^62^. Further evidence on the epigenetic regulation of Gli1 transcriptional activity comes from studies which have documented the recruitment of SAP18, a component of the Sin3 histone deacetylase complex^47–49^, through Sufu to Gli1^45,48^. SAP18 enhances mSin3-HDAC1-mediated transcriptional repression, directing the formation of a repressive complex when tethered on promoters^48^. Besides its involvement in the Hh pathway, SAP18 also functions as a scaffold protein for the formation of a splicing complex and the assembly of other multiprotein complexes via its ubiquitin-like fold^63^. Interestingly, our proteomic analysis identified SAP18 as a new interactor of SET. Furthermore, gene reporter assays showed that SET overexpression relieved the Sufu-mediated inhibition of Gli1 transcriptional activity. Based on these results, we could propose the involvement of SET in the epigenetic network that fine tunes the activity of Gli proteins in response to the Hh signaling gradient through its interaction with SAP18. Further experiments are required to shed light into the underlying mechanisms of SET function in the Hh pathway, facing the major challenges associated with the dynamic nature of Gli-Sufu-SAP18 interactions in the cytoplasm and the nucleus.

## Methods

### Zebrafish experiments

Breeding and maintenance of wild-type zebrafish and 12xGliBS:mCherry-NLS transgenic line were carried out as previously described^36^ in accordance with the European Directive 2010/63 for the protection of animals used for scientific purposes and the Recommended Guidelines for Zebrafish Husbandry^64^ . The experimental protocols described in this study were carried out with zebrafish larvae up to 48 h post fertilization and were approved by the Foundation for Research and Technology-Hellas (FORTH) Ethics Committee. Reverse genetics experiments involving CRISPR/Cas9 were performed and analyzed during embryonic stages of zebrafish development, as described^36^. The study was carried out in compliance with the ARRIVE guidelines (https://arriveguidelines.org).

### Zebrafish embryo microinjections, imaging and analysis

Microinjections of *setb* morpholino (5–7 ng/embryo) were conducted at the one-cell stage of Tg12x_Gli embryos. Monitoring of *setb* morphants and controls was done under stereoscope for scoring phenotypic abnormalities, and brightfield images were obtained at 24 and 48 hpf with DFK2BUC03 camera from The Imaging Source under SMZ1000 stereoscope. mCherry fluorescence (and therefore *Hh* activity) of Tg12x_Gli injected and siblings embryos was monitored at 48 hpf and brightfield and fluorescent images were acquired from injected and control embryos under microscope inverted Leica DMIRE2 with a mounted Hamamatsu ORCA-Flash4.0 camera. In total, 150 embryos were injected. For quantitative image analysis of microscopy images, the open-source software Image J/FiJi (ImageJ 1.52p) (http://imagej.nih.gov/ij)^65^ was used. Pixel intensity was calculated from the captured images by: mean of Region of Interest (ROI)-mean of unstained area (same size as ROI) for each embryo at the region of the brain and the trunk.

### Immunohistochemistry and confocal microscopy

48 hpf embryos were fixed with 4% paraformaldehyde overnight at 4℃. Whole-mount embryos were embedded in 4% agarose and cut in 150 μm thick sections with Leica VT1OOOS Vibratome. Sections were washed 3 times for 15 min with PBT (0.8% Triton X-100 in PBS) and then incubated for 3 h at room temperature in phalloidin-633 (1:300 in PBT) for filamentous actin staining and 20 min at room temperature for DAPI-stained nuclei. Imaging was performed using Leica TCS SP5II upright confocal microscope. Images were capture with LAS AF Software, analyzed in ImageJ Software and presented as maximum projection of a set of z-stacks for each stained tissue section.

### Cell culture and drug treatments

Mouse embryonic fibroblast cells NIH 3T3 and HEK 293 cells were cultured in DMEM (Dulbecco’s Modified Eagle Medium) supplemented with 10% heat inactivated FBS, 100 units/ml penicillin, 100 units/ml streptomycin, 1000 mg/lt glucose and 1 mM L-glutamine. Cells were maintained at 37 °C in a humidified incubator with 5% CO_2_ in the air atmosphere. FTY720 (SML0700, Sigma-Aldrich) was resuspended in sterile H_2_O and used in final concentrations of 5, 7.5 and 10 μM. COG112 (acetyl-RQIKIWFQNRRMKWKKCLRVRLASHLRKLRKRLL-amide) peptide, which was synthesized and purchased from Davids Biotechnologie, was dissolved in PBS and used at a working concentration of 3 μM. The Smo agonist, purmorphamine (Sigma-Aldrich) was dissolved in DMSO and used at a final concentration of 2 μΜ. Pharmacological experiments involving the exposure of cells to SET inhibitors, FTY720 and COG112, were performed in the context of activated Hedgehog signaling. More specifically, NIH 3T3 cells were cultured in DMEM and when cells reached about 60% confluency, the full serum medium was replaced with low serum medium (0.5% FBS). Then, FTY720 or COG112 were added to the medium at different concentrations, and one hour later purmorphamine (2 μΜ) was also added. Cells were collected after 24 h. Protein or total RNA extracts were prepared for further analyses.

### Cilia number measurements

Control NIH 3T3, H4 and H11 cells were serum starved in DMEM containing 0.5% FBS for 24 h and then stained for acetylated tubulin using mouse anti-acetyl-alpha tubulin antibody (clone 6-11B-1 Sigma-Aldrich, 1:2000). The secondary donkey anti-mouse antibody-Alexa Fluor 488 (1:200) was obtained from Invitrogen. The number of the cilia was determined by the acetylated alpha tubulin signals by randomly counting cilia number in three different fields each containing 70–120 cells. For each clone, the experiments were performed in triplicates. Ciliated cells were counted using the open-source software Image J/FiJi (ImageJ 1.52p)^65^.

### Western blot analysis

Cells were cultured in 6-well plates, washed with PBS and resuspended in RIPA buffer (20 mM Tris, 150 mM NaCl, 0.1% Triton X-100, pH 7.4) supplemented with Protease Inhibitor Cocktail (Roche). Protein concentrations were determined by the Bradford Assay (Bio-Rad). For the detection of SET and Gli1, proteins were separated on 10% SDS-PAGE and transferred to nitrocellulose membranes (0.45 μm, GE Healthcare) for 70 min (200 mA). The membranes were cut into two pieces at the 75 kD marker for the detection of Gli1 (upper part) and SET (lower part). The membranes were rinsed with PBS-0.1% Tween 20 (PBST ) and incubated with blocking buffer (5% non-fat milk in PBST) for 1 h at RT and incubated overnight at 4 °C with mouse monoclonal anti-Gli1 (L42B10, Cell Signaling, 1:1000) or for 2 h at RT with mouse monoclonal anti-I2PP2A (F-9 sc-133138 Santa Cruz, 1:2000) or mouse monoclonal anti-α-Tubulin (B-5–1-2 Sigma-Aldrich, 1:4000) to verify equal loading. Membranes were then washed with PBST and incubated with the monoclonal HRP conjugated secondary antibody (1:3000). Proteins were detected using the enhanced chemiluminescence method (ECL, Millipore).

### Luciferase reporter assays

To determine Gli1-mediated transcription, HEK 293 cells were seeded in 24‐well plates and transfected with 360 ng of 12xGli binding site luciferase reporter plasmid (12xGliBS)^42^ and 40 ng of Renilla control reporter plasmid (pPRL-TK), together with 100 ng of pCMV5‐Gli1‐Flag expression construct using jetPEI. The total amount of plasmid DNA in each well, 700 ng, was adjusted with the addition of the pcDNA3.1 vector. After 24 h of plasmid transfection, the cells were lysed and the luciferase activity was measured using Dual-Luciferase Reporter Assay. All experiments were repeated three times and samples were analyzed in triplicates.

### Glutathione S-Transferase (GST) pull down assays

Baculovirus expressed glutathione-S-transferase-tagged SET was prepared according to Bac-To-Bac baculovirus expression system procedure (Invitrogen), as described^15^. To generate GST fusion SAP18, the coding sequence of human SAP18 (isoform 2) was subcloned into EcoRI/XhoI sites of pGEX-6P-3 vector (Pharmacia) in frame with glutathione S-transferase (GST) using the following primers: forward 5′-CCCGAATTCCATGCTCGCTGCAGGGGTCG-3′ and reverse 5′-GGGCTCGAGTTAATATGGTCTCATGCGCCC-3′. The GST-SAP18 protein was expressed in *E. coli* and purified according to standard procedures. For the preparation of cell extracts, HEK 293 cells were transfected with GFP-SET, GFP-SAP18 or GFP expression constructs using jetPEI, as described. 8 × 10^6^ cells were lysed with 150 μl of 50 mM Tris–HCl, pH 7.4, 150 mM NaCl, 1% NP-40, 0.5 mM PMSF (phenylmethylsulfonyl fluoride) supplemented with Protease Inhibitor Cocktail (Roche) and incubated on ice for 5 min. To perform GST-pull down experiments, GST-SET, GST-SAP18 or GST used as control (approximately 2–3 μg each) were immobilized on glutathione-Sepharose beads and the beads were incubated with 150 μg of cell extracts in a total volume of 100 μl for 4 h at 4 °C. Then, the beads were harvested, washed three times with lysis buffer (5 ml total) and resuspended in 25 μl of SDS sample buffer. Bound proteins and the supernatants of the pull down assays were fractionated on 12% SDS–polyacrylamide gels and analyzed by western blotting. GFP-SET and GFP-SAP18 were detected using the goat anti-GFP antibody (SICGEN 1:2000).

### CRISPR/Cas9 technology

Identification and selection of the guide sequences to target *Set* gene, were performed using an online CRISPR Design Tool (http://tools.genome-engineering.org). The search for the most suitable target sites on the genomic sequence of mouse *Set* (Gene ID: 56086, NC_000068.7:30061 996 30072577) was based on the fact that two splice variants, Seta and Setb, are expressed from this gene. Two gRNAs were selected, located in exon 4, the first common exon for both variants. *Set* gRNA1, forward 5′-CACCGCAACTCAGTTCAGTTCAAGT-3′ and reverse 5′-AAACACTTGAACTGAACTGAGTTGC-3′; *Set* gRNA2, forward 5′-CACCGTCTAGTGTCTGCACTGCTTG-3′ and reverse 5′-AAACCAAGCAGTGCAGACACTAGAC-3′. The oligos were synthesized, annealed and cloned into PX459 (Addgene 48139) and pSpCas9(BB)-2A-GFP (PX458, Addgene 48138), respectively. To generate a stable cell line, CRISPR plasmids were introduced into NIH 3T3 cells by transfection with polyethylenimine (PEI). Transfection efficiency was assessed the next day based on GFP expression under a fluorescent microscope. For antibiotic selection, 1.5 μg/ml puromycin was added to the medium and renewed daily. Clonal isolation and expansion were achieved by seeding individual cells in a 96-well plate. Cell growth was monitored and once the formation of single colonies was observed, each monoclonal population was allowed to expand and then transferred to larger culture dishes. To screen for indels genomic DNA was isolated, PCR amplified using primers spanning the target region and subjected to Sanger sequencing (forward 5′-AGTCTGTTTGCAGTTGGTGG-3′ and reverse 5′-CACCTATCAACTGTCTGACTTCAT-3′). Validation of successful gene-editing in selected clones was performed by assessing SET expression levels by western blot analysis using a commercially available antibody (anti-mouse I2PP2A, F-9, sc-133138 Santa Cruz).

### Total RNA isolation and cDNA synthesis

Total RNA was extracted from NIH 3T3 cells (control, treated or CRISPR edited) in three biological replicates using the RNeasy Mini Kit (QIAGEN), according to manufacturer’s protocol. To eliminate genomic DNA contamination, all RNA samples were treated with DNase I (RNase-Free DNase Set). The concentration of the isolated RNA was measured with NanoDrop 2000 spectrophotometer, while its quality and integrity were assessed using the Agilent 2100 Bioanalyzer. One μg of total RNA was used as a template for cDNA synthesis using the PrimeScript RT reagent Kit with gDNA Eraser (Perfect Real Time, TaKaRa), following the manufacturer’s instructions.

### Quantitative real-time PCR

Quantitative Real Time PCR analyses were carried out on a CFX96 Touch Real-Time PCR Detection System (Bio-Rad) with the use of KAPA SYBR FAST qPCR Master Mix (2X) Universal. The reaction mixture constituted of 5 µl of SYBR Green Master Mix (2x), 0.25 µl of each primer (10 μM), 2 µl of diluted cDNA template and 2.5 µl of Nuclease-free water. All biological samples were run in technical triplicates. Cycling conditions consisted of enzyme activation at 95 °C for 3 min and 40 cycles of denaturation at 95 °C for 3 s, annealing at 60 °C for 30 s and extension at 72 °C for 15 s, followed by a melting curve analysis. Mouse *Gapdh* was used as a reference gene. qPCR data analysis, including normalization and relative quantification of gene expression, was performed with the qbasePLUS software (Biogazelle). All primers listed below were designed based on *Mus musculus* nucleotide sequences found in NCBI. *Gli1,* forward 5′-CCAAGCCAACTTTATGTCAGGG-3’ and reverse 5′-AGCCCGCTTCTTTGTTAATTTGA-3′; *Ptch1,* forward 5′-TGCTGTGCCTGTGGTCATCCTGATT-3′ and reverse 5′-CAGAGCGAGCATAGCCCTGTGGTTC-3′; *Ccnd1,* forward 5′-GTTCATTTCCAACCCACCCTC-3′ and reverse 5′-AGAAAGTGCGTTGTGCGGTAG-3′; *Seta,* forward 5′-AGTCTGCGATCCTGCCTCA-3′ and reverse 5′-TGTTCAATTGCTTCTTGCTGTTCT-3′; *Setb,* forward 5′-CCGACGAGACCTCAGAAAAAGA-3′ and reverse 5′-AAAATGGTTGGCGGAGTTTG-3′. *Gapdh,* forward 5′-AGGTCGGTGTGAACGGATTTG-3′ and reverse 5′-GGGGTCGTTGATGGCAACA-3′.

### Statistical analysis

Statistically significant changes in gene expression were determined using the One-way ANOVA test followed by Tukey–Kramer post hoc analysis for pairwise comparisons, considering *p < 0.05, **p < 0.01, ***p < 0.001. Statistically significant difference of pixel intensity was determined using two-tailed Student’s t-test, using GraphPad Prism 5.0. *p* value was considered significant ***p < 0.001.

## Supplementary Information

Supplementary Information.
