# Supplementary material for: Targeting of SET/I2PP2A oncoprotein inhibits Gli1 transcription revealing a new modulator of Hedgehog signaling
Source: Sci Rep. 2021 Jul 6;11:13940. doi: 10.1038/s41598-021-93440-0 (PMC8260731; doi:10.1038/s41598-021-93440-0)

Targeting of SET/I2PP2A oncoprotein inhibits Gli1 transcription revealing a new modulator of Hedgehog signaling

Authors

Iliana Serifi[1,2], Simoni Besta[1], Zoe Karetsou[1], Panagiota Giardoglou[3], Dimitris Beis[3], Pawel Niewiadomski[4] and Thomais Papamarcaki[1,2]*

Affiliations

[1]Laboratory of Biological Chemistry, School of Health Sciences, Medical Department, University of Ioannina, Greece

[2]Foundation for Research and Technology-Hellas, Institute of Molecular Biology and Biotechnology, Department of Biomedical Research, Ioannina, Greece

[3]Developmental Biology, Center for Clinical, Experimental Surgery and Translational Research, Biomedical Research Foundation Academy of Athens, Athens 11527, Greece

[4]Centre of New Technologies, University of Warsaw, Warsaw, Poland

Supplementary figures

Figure 1S. Hh signaling is reduced in Tg12x_Gli transgenic zebrafish embryos injected with *seta/b* morpholino (MOab).

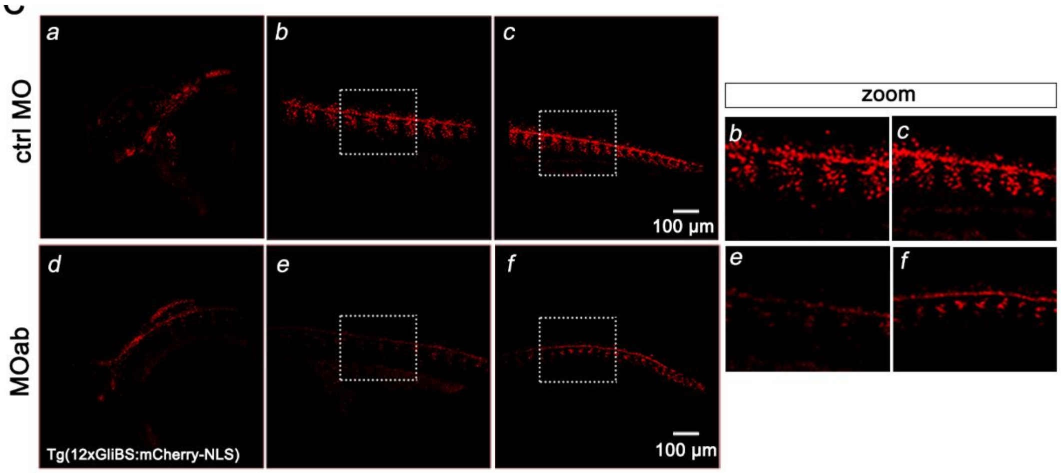

Figure 1S. One- to two-cell stage Tg12x_Gli transgenic zebrafish embryos were injected with 7.5 ng of *seta/b* translation blocking morpholino (MOab) and examined under a confocal fluorescence microscope (n=62). The squares show enlargement of the trunk areas which display decreased mCherry fluorescence intensity. Note the disrupted morphology of the somites in the marked regions.

Figure 2S. Uncropped/Unprocessed Figures

Fig. 3A

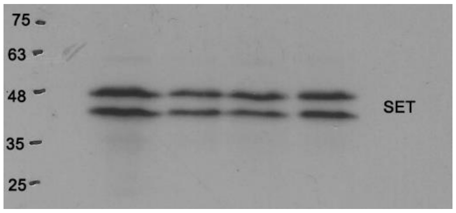

Fig.3A

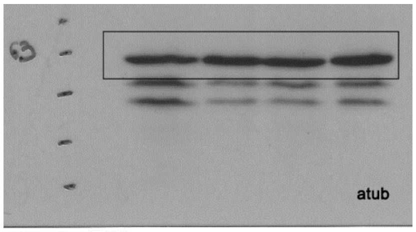

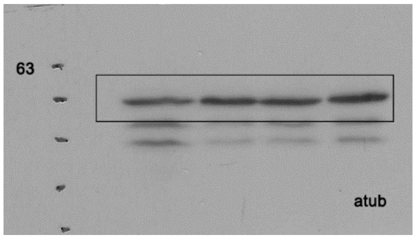

Second exposure

Fig. 3C

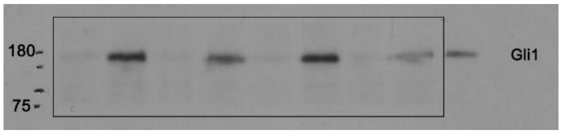

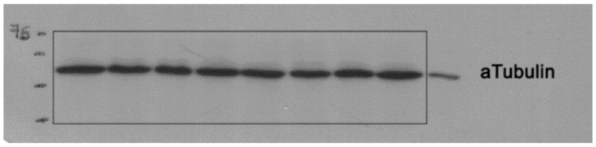

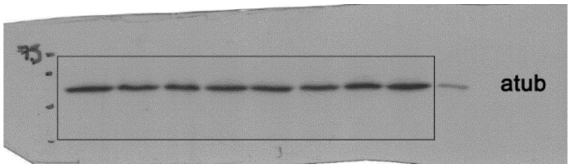

Second exposure

Fig. 4

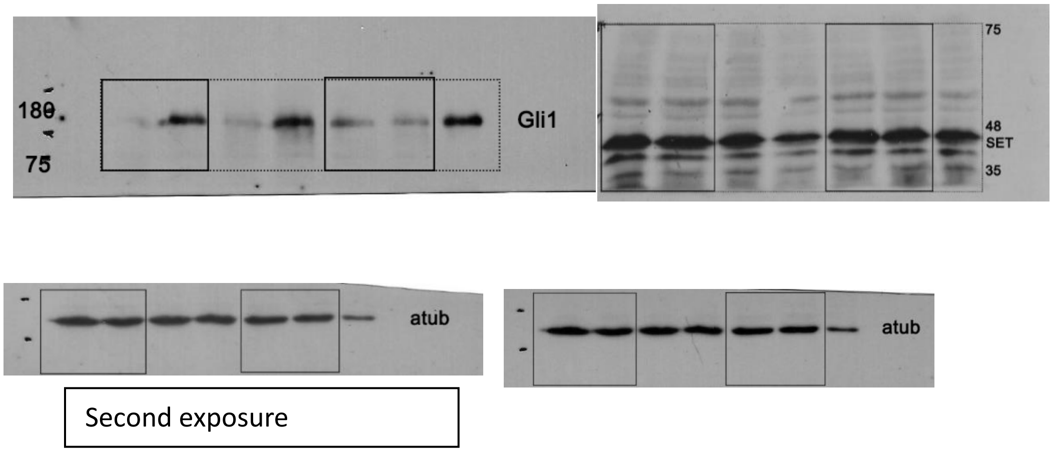

Figure 5

Fig. 5C

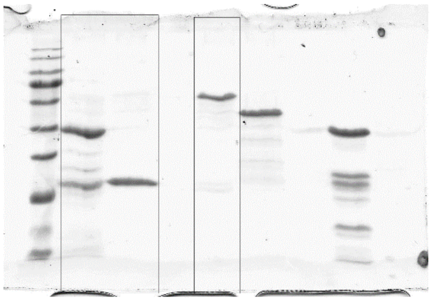

Fig. 5D

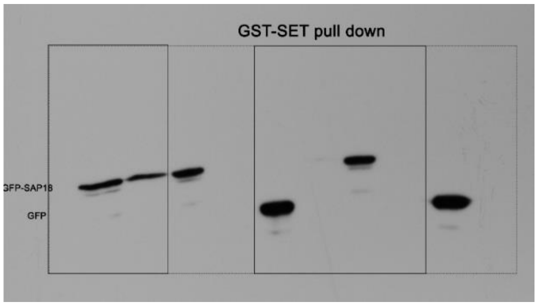

Fig. 5E

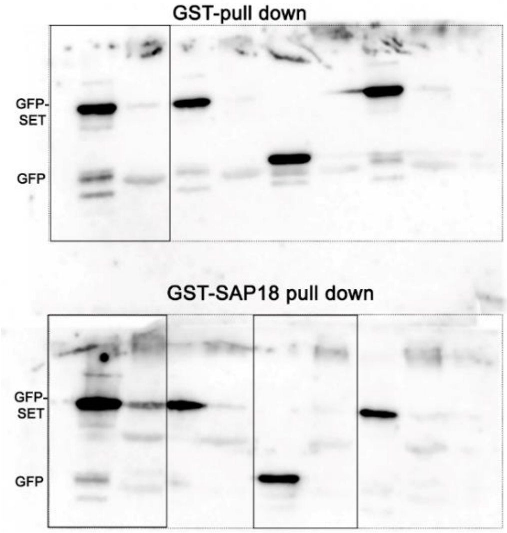

Supplement: Supplementary file 1 — Supplementary Information. [file 41598_2021_93440_MOESM1_ESM.pdf]
